# Challenges in Preventive Practices and Risk Communication towards COVID-19: A Cross-Sectional Study in Bangladesh

**DOI:** 10.3390/ijerph18179259

**Published:** 2021-09-02

**Authors:** Farah Naz Rahman, Md Al Amin Bhuiyan, Kabir Hossen, Hafiz T. A. Khan, AKM Fazlur Rahman, Koustuv Dalal

**Affiliations:** 1 Office of the Executive Director, Centre for Injury Prevention and Research Bangladesh (CIPRB), Dhaka 1206, Bangladesh; farah.naz@ciprb.org (F.N.R.); al_amin_prime@yahoo.com (M.A.A.B.); kabir@ciprb.org (K.H.); fazlur@ciprb.org (A.F.R.); 2 College of Nursing, Midwifery and Healthcare, University of West London, London W5 5RF, UK; htakhan@yahoo.com; 3 National Public Health Advisory Committee on COVID-19 Management, Directorate General of Health Services (DGHS), Dhaka 1212, Bangladesh; 4 Division of Public Health Science, School of Health Sciences, Mid Sweden University, 851 70 Sundsvall, Sweden

**Keywords:** Bangladesh, COVID-19, pandemic, protective behavior, risk communication

## Abstract

Bangladesh recently experienced a COVID-19 second wave, resulting in the highest number of new cases and deaths in a single day. This study aims to identify the challenges for COVID-19 preventive practices and risk communications and associated factors among Bangladeshi adults. A cross-sectional survey was conducted between December 2020 and January 2021 involving 1382 Bangladeshi adults (aged ≥ 18-years) in randomly selected urban and rural areas from all eight divisions in Bangladesh. Descriptive data analysis was conducted to highlight the challenges for preventive practices and risk communications for COVID-19. Multiple logistic regression analysis was used to determine the sociodemographic groups vulnerable to these challenges. Lack of availability of protective equipment (44.4%), crowded living situations/workspaces (36.8%), inadequate information on the proper use of protective measures (21.9%), inadequate handwashing and sanitation facilities (17.6%), and negative influences on family/friends (17.4%) were identified as barriers to COVID-19 preventive practices. It was also found that males (OR = 1.3, 95% CI = 1.01, 1.7), rural residents (OR = 1.5, 95% CI = 1.2, 2), respondents with a low level of education: no schooling vs. ≥higher secondary (OR = 3.5, 95% CI = 2.3, 5.2), primary vs. ≥higher secondary (OR = 2.5, 95% CI = 1.7, 3.8), respondents engaged in agricultural (OR = 1.7, 95% CI = 1.2, 2.4), laboring (OR = 3.2, 95% CI = 2, 5), and domestic works (OR = 1.6, 95% CI = 1.07, 2.5), and people with disabilities (OR = 1.7, 95% CI = 1.1, 2.6) were all likely to have difficulty in practicing effective COVID-19 protective behaviors. Respondents’ education and occupation were significant predictors of inadequate understanding of COVID-19 risk communications and was identified as a problem among 17.4% of the respondents. A substantial percentage of Bangladeshi adults have difficulty practising COVID-19 protective behaviours and have poor comprehension of risk communications, particularly in rural areas and among those with low education. This research can aid policymakers in developing tailored COVID-19 risk communications and mitigation strategies to help prevent future waves of the pandemic.

## 1. Introduction

Following the detection of the first case of COVID-19 in Bangladesh on 8 March 2021, the country now has about 0.8 million cases with 12 thousand deaths [1]. Bangladesh experienced a surge in infections from June to August 2020, marking the first wave of the virus. Several containment measures were applied to control the situation, including a countrywide lockdown and travel and social activities restrictions. Risk communication strategies were also developed and deployed in the country as part of the National Pandemic Preparedness and Response Plan (NPPRP) [2]. The NPPRP defines ‘risk communication’ as a strategy for equipping individuals and communities with the knowledge and skills needed to prevent the spread of COVID-19 through informed individual decisions and behavior change. The goal of risk communication is to provide people with life-saving information while also ensuring that the information is internalized so that a change in people’s behavioral practices can be facilitated [3]. In addition to these measures, information on COVID-19 was widely disseminated, and advocacy for practising WHO-recommended preventive behaviours was presented via electronic, print, and social media.

Despite these ongoing efforts, the second wave of COVID-19 started in Bangladesh during the second week of March 2021 [4]. Expert opinion suggests that inadequate preventive measures such as wearing masks, hand washing, and social distancing contributed to the emergence of this second wave [5]. Concerns have been raised about the difficulty of implementing recommended preventive behaviours such as “maintaining social distance” and “avoiding social gatherings” in a densely populated country like Bangladesh [6]. In addition, although the prevalence of mask use has improved over the year, a substantial number of people are still wearing them inappropriately [7,8]. A considerable number of people also had inadequate access to protective equipment such as masks, gloves, and hand sanitizer [9,10]. Furthermore, barriers to healthcare, health safety, and health promotion measures have disproportionately impacted persons with disabilities during COVID-19 in Bangladesh, making them a vulnerable group to the pandemic’s consequences [11,12,13].

Several studies in Bangladesh that assessed the knowledge level of respondents regarding COVID-19, also found sociodemographic factors such as age, gender, residence, and education, to be significant predictors of inaccurate or low COVID-19 knowledge [14,15,16]. Furthermore, different socioeconomic groups in Bangladesh have different levels of understanding of generalized information on COVID-19 precautions. Some people are having difficulty understanding terms such as “social distance” and “quarantine,” which do not have a proper translation in the native language [10,17]. Misconception on COVID-19 has been another predominant challenge in the risk communication strategies around the world. An exploratory study in Canada revealed that the participants perceived public health messages on COVID-19 as conflicting, with perceptions varying by age-group [18]. Another large survey with a diverse sample in USA found that different ethnic groups interpret and endorse COVID-19 risk communication messages differently [19]. However, such studies are limited in the low-income countries, including Bangladesh. The weakness in risk communication campaigns became apparent in Bangladesh, when about 200 online rumours related to COVID-19 spread across the country [20]. Moreover, in a cross-sectional study in Bangladesh, more than half of the respondents were found to have misconceptions about COVID-19, with education being a significant determinant [21]. The differences in perception and misconception across different sociodemographic groups highlight the need to investigate the understanding of uniformly distributed COVID-19 risk communication messages among various groups in Bangladesh.

There is growing evidence that preventive behaviour practices influence COVID-19 transmission, and such practices are influenced by risk communications [22,23,24]. As a result, the factors that limit these activities play an important role in the resurgence of COVID-19 infection. It is imperative to identify the challenges associated with preventive practice and risk communication, as well as their determinants, in order to optimize and strengthen current strategies. Although some anecdotal reports highlighted the barriers to practising preventive measures and understanding risk communications, any empirical evidence of this is still unavailable. Therefore, the purpose of this study is to investigate the challenges in practising preventive behaviour and risk communications for COVID-19 in a low-resource country setting to help prevent any future waves of this virus and similar diseases.

## 2. Materials and Methods

A cross-sectional survey was conducted from December 2020 to January 2021 with data collected from adults in Bangladesh aged 18 years and above. A multi-stage cluster randomized sampling technique was used to recruit a total of 1382 participants from both urban and rural regions. 

**Figure 1:**
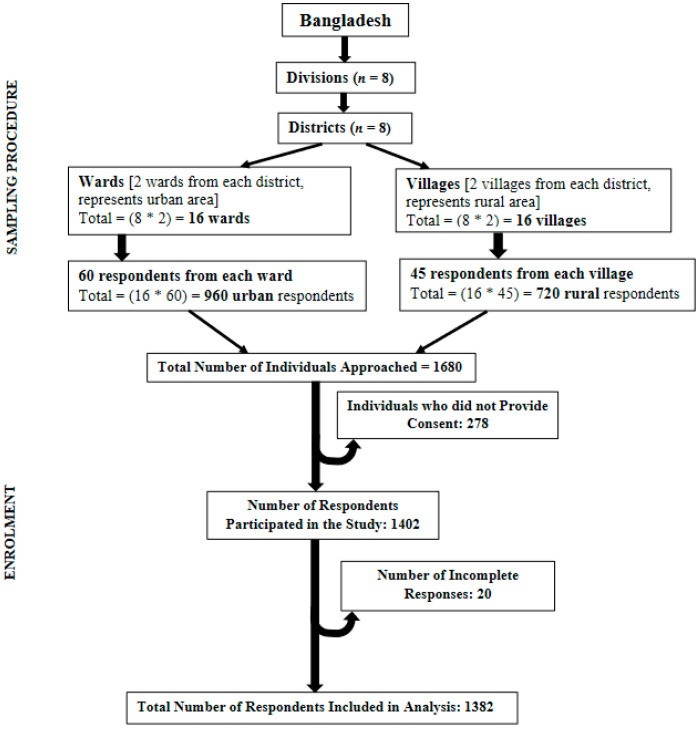
Sampling technique and steps to include respondents in analysis in the cross-sectional study on challenges in preventive practices and risk communication for COVID-19.

Bangladesh has eight major administrative units called divisions: Dhaka, Chattogram, Mymensingh, Rajshahi, Khulna, Barishal, Sylhet, and Rangpur. One district was selected from each division, giving eight districts: Dhaka, Coxs’ Bazaar, Patuakhali, Khulna, Sirajganj, Habiganj, Sherpur, and Rangpur. Two wards (elective units of a city corporation) were randomly selected from each district headquarter or city corporation representing urban regions. Alongside these, two villages were randomly chosen from each district to represent respondents from rural regions and a further 60 households were randomly selected from each ward and 45 households selected from each village. The prevalence of the COVID-19 virus is higher in urban areas and so more households were targeted from these areas than from rural areas. One eligible respondent from each household was randomly approached for consent to take part in the study. Eligibility comprised Bangladeshi nationals ≥18 years of age and lived in the household for at least one year. Following this procedure, 1680 adults were approached and of this total, 278 (urban = 159, rural = 119) did not consent to participate and 20 respondents provided incomplete responses. Excluding the incomplete responses, data from a total of 1382 respondents were included in the analysis. Figure 1 presents the sampling technique and the procedure for including respondents.

### 2.1. Data Collection and Ethical Considerations

A total of 10 data collectors (DCs) were recruited and trained to gather data from households. The first group of households were chosen from the approximate geographical centre of one ward or village and then the DCs visited households in an anticlockwise direction. Informed written consent was taken from each respondent, and data collected using a pretested semi-structured questionnaire. The DCs maintained all necessary COVID-19 safety precautions (e.g., personal protective equipment—gloves, mask, hand sanitizer—and social distancing) while conducting face-to-face interviews. Ethical approval for this study was obtained from the institutional ethical review committee of the Centre for Injury Prevention and Research Bangladesh (CIPRB) [Ref: ERC/CIPRB/08052020]. The study adhered to all ethical principles, including the Directorate General of Health Services Bangladesh (DGHS) guidelines for researching the pandemic.

### 2.2. Variables and Statistical Analysis

Age, sex, education, occupation, residence location, and disability were included as sociodemographic variables and these were then categorized as age groups (18–30, 31–45, 46–60, 60+ years), sex (male, female), education (no formal education, 1–5 years of schooling = primary, 6–10 years of schooling = secondary, >10 years of schooling = higher secondary and above), occupation (domestic work, service, business, agriculture, labouring work), residence location (urban, rural), and disability (present, absent). Respondents were asked about their source for receiving COVID-19 information and their level of understanding of it by use of a five-point Likert scale: ‘understands all of it/understands most of it/understands some of it/understands little/did not understand at all, and whether they wanted more information on some aspects of COVID-19 (transmission, symptom, precaution, test, treatment, vaccine). Respondents were also asked if they faced any difficulties in practising the WHO recommended preventive behaviours during the last month, to which they could respond ‘yes/no’. Information on the cause of difficulties in practising preventive measures was also gathered.

The five-point Likert scale responses on levels of understanding of COVID-19 information were converted to a binary outcome variable with categories—‘good understanding’ and ‘inadequate understanding’. For the new variable, ‘understands all of it’ and ‘understands most of it’ were grouped under ‘good understanding’, and the remaining three responses were grouped to present ‘inadequate understanding’. Multiple logistic regression analysis was then used to help identify the sociodemographic predictors of ‘inadequate understanding’ of COVID-19 information, where age, sex, education, occupation, and residence location were used as independent variables. Similarly, multiple logistic regression analysis helped to determine the risk groups that faced challenges in preventive practices. In this analysis, education, occupation, residence location, and disability were used as independent variables, and ‘whether they faced any difficulty in preventive practice (yes/no)’ was used as an outcome variable. All the assumptions for regression analysis were met and statistical significance was considered at *p*-value < 0.05. IBM SPSS Statistics v24 software (International Business Machines Corporation, New York, NY, USA) was used for analyzing all quantitative data.

## 3. Results

### 3.1. Sociodemographic Characteristics of the Respondents

A total of 1382 Bangladeshi adults aged 18 years and above participated in the study. The sociodemographic characteristics of the respondents are presented in Table 1. 

As Table 1 shows, most of those that responded were in the younger and middle-aged groups (between 18 to 45 years of age), with older adults (60+ years) making up around 7% of the total. The proportion of male and female respondents was nearly equal, with a male-to-female ratio of 1.06:1. Approximately 17% of respondents had no institutional education, while one-third (34.1%) had a higher level of education (higher secondary and above). Urban residents comprised 57.4% of the total respondents. Those working in agricultural and other labouring pursuits made up around 40% of all study participants, with 27.5% engaged in business activities, and both service holders and domestic workers accounted for 16.1% of the total. Around 11% of respondents reported having some form of physical disability.

### 3.2. Challenges in Practicing COVID-19 Preventive Behavior among Bangladeshi Adults

**Figure 2:**
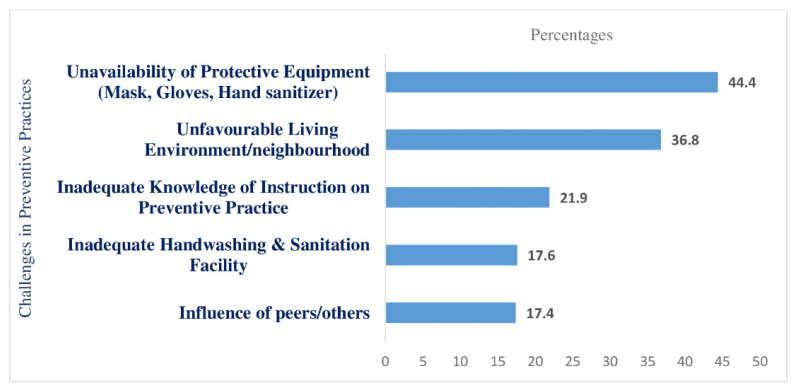
Challenges in preventive practices for COVID-19 faced by respondents.

Nearly 71% of respondents indicated that they faced difficulties in practising the recommended COVID-19 preventive behaviours. Figure 2 presents the nature of these challenges.

Unavailability of protective equipment for COVID-19 tops the challenges list for respondents in adopting preventive practices. Almost 45% reported having insufficient protective equipment such as masks, gloves, soap, and hand sanitizers. More than one-third (36.8%) also stated that their efforts were hampered by crowded or congested living conditions and in the workplace and 17.6% cited inadequate handwashing and sanitation facilities as barriers to practising preventive measures. Nearly 22% of respondents said that inadequate knowledge of instructions regarding protective measures such as proper use of masks, hand washing techniques, and social distancing had been challenging. In addition, negligence in using protective measures by other family members, friends, and residents discouraged approximately 17% of respondents from engaging in preventive practices themselves.

### 3.3. Factors Associated with Challenges in COVID-19 Preventive Practices

**Figure 3:**
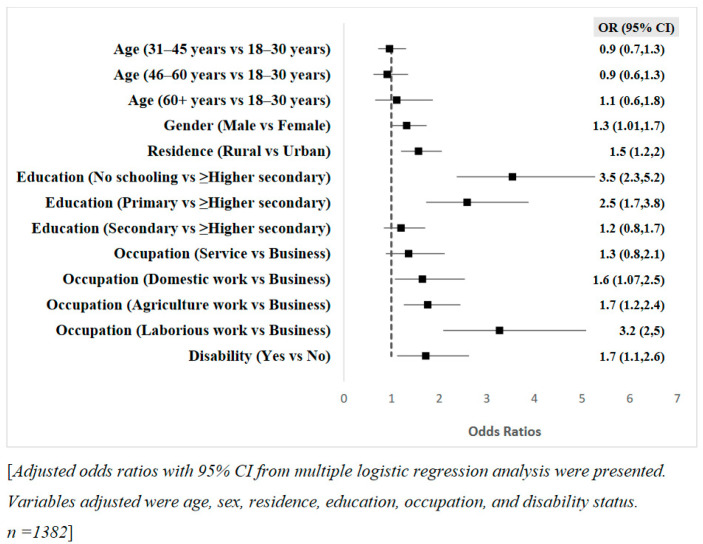
Predictive factors for experiencing challenges in COVID-19 preventive practices among Bangladeshi adults.

A multiple logistic regression analysis was used to explore the relationship between the sociodemographic characteristics of respondents and their likelihood of experiencing difficulties in pursuing preventive practices for COVID-19. Figure 3 presents the sociodemographic determinants of challenges in COVID-19 preventive practices.

Gender, residence, education, occupation, and disability were significantly associated with the likelihood of experiencing difficulties in COVID-19 preventive practices among Bangladeshi adults. Males were 1.3 times more likely than females to face difficulties and those respondents in rural areas had 1.5 times higher odds of experiencing challenges than did respondents in urban areas. Respondents that did not have any schooling and those with primary education were respectively 3.5 and 2.5 times more likely to have difficulties practising preventive behaviours compared to respondents with an education level of higher secondary or above. Additionally, domestic workers, agricultural workers, and day labourers were 1.6, 1.7, and 3.2 times more likely to face problems than those working in the business. Challenges in COVID-19 preventive practices were 1.7 times higher among persons with disabilities.

### 3.4. Challenges in Risk Communications for COVID-19 among Bangladeshi Adults

**Figure 4:**
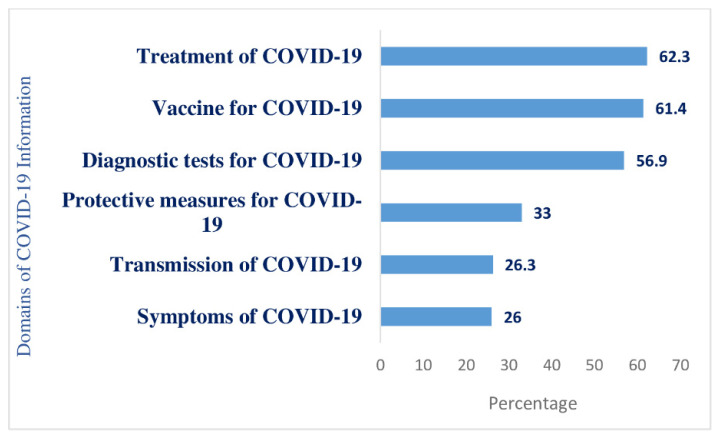
Proportion of respondents with inadequate information according to COVID-19 domains.

Almost all (98.8%) of respondents said they had been exposed to various COVID-19 awareness campaigns, including information via electronic, print, and social media, community distribution of leaflets, miking, and information from health workers or community leaders. Respondents shared their need for more information on specific areas related to COVID-19, as Figure 4 shows.

Most respondents (62.3%) said they had inadequate information on treatments for COVID-19, including information on dedicated healthcare facilities and treatment from home procedures. About 60% had inadequate information on the vaccine, including the registration procedure, safety, and effectiveness and more than half (56.9%) reported having inadequate information on diagnostic tests. Around one-third (33%) of respondents wanted more information about protective measures and instructions on their proper use and just over 26% wanted more information about symptoms and the transmission modality of COVID-19.

### 3.5. Determinants of Inadequate Understanding of COVID-19 Information among Bangladeshi Adults

Respondents shared their level of comprehension of the COVID-19 information they have received on a five-point Likert scale (understands all of it/understands most of it/understands some of it/understands little/did not understand at all). The majority of respondents (66.3%) stated that they understood most of the information received, 16% said they understood it, and 4.1% stated they understood some of it. However, approximately 11% reported having little understanding of the received information, and 2.2% of having no understanding. The five Likert scale responses were converted to a binary outcome variable—‘good understanding/inadequate understanding’ (see methodology), and multiple logistic regression was carried out. Adjusted odds ratios from the multiple logistic regression analysis, predicting the effects of sociodemographic variables on the level of understanding of COVID-19 information among Bangladeshi adults, is presented in Table 2.

Education and occupation were significantly associated with the level of understanding of COVID-19 information among respondents. Low education was associated with a low level of understanding and inadequate understanding was nearly 13.5 times higher among respondents without any institutional education than those with a higher secondary or higher education level. Inadequate understanding of COVID-19 information among respondents with primary and secondary education was seven times and four times higher than those with an education level of higher secondary or above. Agricultural workers and day labourers were approximately twice as likely as businesspeople to have an inadequate understanding of COVID-19 information. Domestic workers were also 1.7 times more likely to have an inadequate understanding than those engaged in business.

## 4. Discussion

The first time our study using the context in Bangladesh has tried to provide empirical evidence on the challenges in preventive practices and risk communications for COVID-19 among Bangladeshi adults around the time of the second wave of the pandemic. The study analyzed data from face-to-face interviews conducted in rural and urban areas across all eight divisions of Bangladesh, allowing for greater generalizability of the findings. Limited availability of protective equipment such as masks, gloves, hand sanitizer, and crowded living situations and workspaces were the barriers for COVID-19 preventive practices among about 40% of the respondents. Additionally, male respondents, rural residents, respondents with a low level of education, those engaged in agricultural, labouring, and domestic work, and people with disabilities were more likely to have difficulty practising COVID-19 protective behaviours. Although almost all of the respondents had been exposed to some form of COVID-19 awareness campaign, 17.4% had an inadequate understanding of the information they received. Furthermore, a large number of respondents reported a lack of knowledge about COVID-19 diagnostic tests, treatment, and vaccines. The education and occupations of respondents were significant predictors of inadequate understanding of COVID-19 risk communications.

The respondents’ top three preventive practice challenges were lack of protective equipment, crowded living spaces, workspaces, and neighbourhoods, and inadequate knowledge on the proper use of protective measures. These findings are reflected in an ongoing study in Bangladesh that has monitored mask use among northern Dhaka dwellers and revealed improper mask use among 25% of the citizens, indicating a lack of knowledge on their proper use [25]. This ongoing study also tracked improper social distancing on 14 June 2021 among 53% of the citizens. In addition, a large randomized controlled trial (RCT) in Bangladesh involving 350,000 people considered the unavailability of masks and lack of knowledge on their proper use as barriers to preventive practice and found that no-cost mask distribution and sharing information on wearing them through electronic and print media increased better practice among community people [26]. 

The findings of this current study are also consistent with the findings of an exploratory study conducted among garment workers in Bangladesh that identified community living in close proximity as a barrier to maintaining social distance [27]. This current study further identified inadequate sanitation facilities and negative influences of family/friends as barriers to preventive practices for COVID-19. Along a similar line, experts have highlighted the lack of sanitation facilities as a potential barrier to COVID-19 preventive practices in Bangladesh [6], and a large RCT identified modelling and endorsement by trusted leaders as a useful measure to increase mask use among community people [26]. 

Sociodemographic groups that are more likely to face barriers, and be more vulnerable in practising COVID-19 protective behaviours, were identified as male, rural residents, and those with a low level of education. Our findings are in line with several other studies conducted in Bangladesh on COVID-19 prevention practices that identified significantly lower practices among males, rural residents, and those with low education [16,28,29]. Bangladeshi men tend to be very outgoing and are often the sole wage earners of the family, a situation that forces them to work during the restriction period and exposes them to crowded workplaces and social gatherings during the pandemic. Alongside this, rural residents have a lower level of education and come from a poorer socioeconomic background than urban residents. This limits the ability of rural residents to access or afford COVID-19 protective equipment and their ability to understand instructions on how to use them. Large families living in congested areas are also common in rural areas, making social distancing impossible [30,31]. 

This situation also applies to agricultural workers, day labourers, and domestic workers who are from low socioeconomic groups and have a low level of education and were also found to be more vulnerable to barriers in COVID-19 preventive practices in this current study. Furthermore, people with disabilities were found to be more vulnerable to the challenges of protective behaviours in this study. Another study reviewed the situation of those with disabilities in Bangladesh during the pandemic, and identified marginalization and the constant need for care from others as barriers to their safety from COVID-19 [11].

Despite widespread dissemination of COVID-19 information as part of the NPRP, approximately 60% of respondents in this study had insufficient knowledge of COVID-19 diagnostic tests, treatment, and vaccines. Bangladesh has been running very low on COVID-19 diagnostic tests, with only about 5000 tests per million people for a population of over 160 million [32]. The country has been relying on passive testing by the population rather than actively screening for cases. A lack of knowledge about diagnostic facilities among the general population, therefore, may have contributed to low testing coverage and, as a result, limited the case detection procedure. Furthermore, since the beginning of the pandemic, several reports have highlighted the difficulty people have in getting COVID-19 treatment in the country [20,33]. The separation of COVID-19 management from regular hospitals to dedicated centres confused the general public, indicating a lack of readily available information. Besides that, the national COVID-19 management guidelines recommend that patients with mild symptoms should be treated at home with physician consultation via telemedicine [34]. However, rural residents, people with low socioeconomic and educational backgrounds, and those from disadvantaged communities had difficulty adhering to self-quarantine, isolation, and home treatment procedures [31,32], further pointing to a weakness in the COVID-19 information campaigns.

Additionally, inadequate vaccine information among respondents is consistent with the findings of another cross-sectional survey that found vaccine refusal and hesitancy among one-fourth of their participants [35]. About 21% of the respondents in this current study also reported having insufficient information on protective behaviours that potentially contributed to improper use of masks, personal protective equipment (PPE), and faulty hand washing techniques [7,36,37]. Nearly one-fifth of respondents were also found to have an inadequate understanding of COVID-19 information that was more common among people with a low level of education and those working in agricultural, labouring, and domestic jobs. Although no studies evaluating the level of understanding of COVID-19 risk communications are available, a few studies have found an association between low education and lower knowledge of COVID-19 among the Bangladeshi population [28,29]. Furthermore, the vulnerable occupation group, particularly day labourers and agricultural workers, faces intersectional disadvantage because of their low socioeconomic and educational backgrounds, making existing risk communication strategies less comprehensible. Qualitative studies exploring the specific causes could provide better understanding. However, the current study has indicated the issues and important points for the policy makers for necessary actions. 

### Limitations and Directions for Future Research

The study findings have a few limitations. Socioeconomic information could not be collected from respondents and meant that the variation in challenges regarding COVID-19 preventive practices and risk communications across socioeconomic groups could not be determined. However, the variation across related social determinants of health, such as education and occupation, was investigated and risk groups were identified whose economic status could provide insights into economic variability. In addition, the underlying causes of these challenges among different groups could not be investigated due to data limitations. For instance, the data do not adequately represent marginalized groups such as indigenous peoples and urban slum dwellers that meant it was not possible to determine how the challenges were distributed among these communities.

Future exploratory research can look in-depth at the causes of challenges and barriers in COVID-19 preventive practices and risk communications among various sociodemographic groups and how these factors influence the transmission of COVID-19 among them. Further research with a more inclusive approach could also explore these challenges among marginalized communities in Bangladesh. Moreover, building on the evidence from this study, future research may investigate how to mitigate these challenges and barriers through developing intervention strategies.

## 5. Conclusions

This study identified the unavailability of protective equipment and crowded living spaces as significant barriers to practising COVID-19 protective behaviours and identified those sociodemographic groups that are more likely to face these barriers. This evidence can help policymakers develop intervention strategies such as the free distribution of masks and other protective equipment, particularly for vulnerable groups. It also emphasizes the need for developing culture- and context-specific alternative strategies for people whose socioeconomic circumstances do not allow them to maintain recommended protective behaviours such as “social distancing” and “frequent handwashing.” Persons with disabilities were identified as a vulnerable group for the challenges in COVID-19 preventive practices in this study, highlighting the importance of focusing on the needs of marginalized communities through targeted research and programs. Furthermore, inadequate information regarding the proper use of protective measures was a critical challenge in both preventive practices and risk communications for COVID-19. Therefore, strengthening the ‘how to’ component of risk communication campaigns is recommended while advocating for COVID-19 protective behaviours.

Additionally, an insufficient flow of information was identified in vital COVID-19 domains such as diagnostic tests, treatment, and vaccines for the virus. This calls for optimization of the national COVID-19 awareness campaign, risk communications, and vaccination campaign strategies. Moreover, the lower comprehension of the COVID-19 awareness campaign among agricultural workers, day labourers, and people with low education levels highlights the necessity of developing risk communication messages tailored to people’s social context and need.

## Figures and Tables

**Table 1:** Sociodemographic Characteristics of the Bangladeshi Adults Participated in the Cross-sectional Study on Challenges in Preventive Practices and Risk Communication for COVID-19 (*n* = 1382).

Characteristics of Respondents	Measurement of Variables	Number (*n*)	Percentage (%) Total
Age	Age 18 to 30 years	449	32.5
	Age 31 to 45 years	571	41.3
	Age 46 to 60 years	255	18.5
	Age 60+ years	107	7.7
Gender	Male	712	51.5
	Female	670	48.5
Disability	Yes	151	10.9
	No	1231	89.1
Residential Location	Urban	792	57.4
	Rural	590	42.6
Education	No Literacy	238	17.2
	Primary	348	25.2
	Secondary	325	23.5
	Higher Secondary and Above	471	34.1
Occupation	Business	380	27.5
	Service	223	16.1
	Domestic Work	222	16.1
	Agriculture	149	10.8
	Laborious Work (Rickshaw Puller, Day Labourers etc.)	408	29.5

**Table 2:** Determinants of Inadequate Understanding of COVID-19 Information among Bangladeshi Adults.

Independent Variables	Outcome Variables		Sig.	OR	95% CI
	**Inadequate Understanding *n* (%)**	**Good Understanding *n* (%)**			
Age					
18–30 years	71 (15.8%)	378 (84.2%)		1	
31–45 years	104 (18.2%)	467 (81.8%)	0.68	0.92	0.64–1.33
46–60 years	49 (19.2%)	206 (80.8%)	0.58	0.87	0.55–1.40
60+ years	16 (15%)	91 (85%)	0.05	0.52	0.27–1.00
Gender					
Female	116 (17.3%)	554 (82.7%)		1	
Male	124 (17.4%)	588 (82.6%)	0.51	1.11	0.80–1.56
Residence					
Urban	126 (15.9%)	666 (84.1%)			
Rural	114 (19.4%)	474 (80.6%)	0.64	1.07	0.78–1.47
Education					
Higher Secondary and Above	20 (4.2%)	51 (95.8%)		1	
Secondary	46 (14.2%)	279 (85.8%)	0.00	4.05 **	2.30–7.15
Primary	84 (24.1%)	264 (75.9%)	0.00	6.99 **	4.02–12.14
No Schooling	90 (37.8%)	148 (62.2%)	0.00	13.47 **	7.52–24.12
Occupation					
Business	39 (10.3%)	341 (89.7%)	-	1	-
Service	19 (8.5%)	204 (91.5%)	0.05	1.88	1.00–3.52
Domestic work	35 (15.8%)	187 (84.2%)	0.03	1.79 *	1.03–3.10
Agriculture work	35 (23.5%)	114 (76.5%)	0.01	1.97 *	1.13–3.43
Laborious work	112 (27.5%)	296 (72.5%)	0.00	2.39 **	1.55–3.67

[Adjusted odds ratio (OR) from multiple logistic regression analysis illustrating the likelihood of low to moderate understanding of COVID-19 information across sociodemographic variables. Outcome variables were categorized as ‘inadequate understanding = 1’ and ‘good understanding = 0’. Variables adjusted were age, gender, residence, education, occupation. The first category under each independent variable was considered the variable’s reference category. * *p* < 0.05, ** *p* < 0.01; *n* = 1382].

## Data Availability

Deidentified data is publicly available at 10.6084/m9.figshare.14794326 (accessed on 10 July 2021).

## References

[1] Worldometer Bangladesh COVID: 794,985 Cases and 12,480 Deaths. https://www.worldometers.info/coronavirus/country/bangladesh/.

[2] Government of Bangldesh National Preparedness and Response Plan for COVID-19, Bangladesh. https://reliefweb.int/report/bangladesh/national-preparedness-and-response-plan-covid-19-bangladesh.

[3] Ministry of Health and Family Welfare (2020). Bangladesh Preparedness and Response Plan for COVID-19.

[4] Khan M.K. Second Wave of COVID-19 in Bangladesh and Concerns. https://www.thedailystar.net/health/news/second-wave-covid-19-bangladesh-and-concerns-2006337.

[5] Health Minister Blames Negligence for Rise in Coronavirus Cases. https://www.dhakatribune.com/bangladesh/2021/04/03/health-minister-blames-negligence-for-rise-in-coronavirus-cases.

[6] Anwar S., Nasrullah M., Hosen M.J. (2020). COVID-19 and Bangladesh: Challenges and How to Address Them. Front. Public Health.

[7] Rahman M.H. (2020). Inappropriate use and disposal of face masks may promote the spread of COVID-19 in Bangladesh. Popul. Med..

[8] Khyum K. COVID-19, Dhaka Division Worst at Wearing Face Masks. https://www.dhakatribune.com/health/coronavirus/2020/07/28/dhaka-division-worst-in-bangladesh-for-wearing-masks.

[9] Banik R., Rahman M., Sikder T., Gozal D. (2020). COVID-19 in Bangladesh: Public awareness and insufficient health facilities remain key challenges. Public Health.

[10] Hasanul M., Siam B., Hasan M.M., Tashrif S.M., Hasinur M., Khan R., Raheem E., Hossain M.S. (2021). Insights into the first seven-months of COVID-19 pandemic in Bangladesh: Lessons learned from a high-risk country. Heliyon.

[11] Kibria G., Islam T., Miah S., Ahmed S., Hossain A. (2020). Barriers to healthcare services for persons with disabilities in Bangladesh amid the COVID-19 pandemic. Public Health Pract..

[12] Hasan M.T., Das A.S., Ahmed A.I., Chowdhury A.M.R., Rashid S.F. (2021). COVID-19 in Bangladesh: An especially difficult time for an invisible population. Disabil. Soc..

[13] Uddin T., Mohammad H.T., Siddiquee N. (2021). COVID-19 Pandemic: Ethical and medical issues arising for people with disability in Bangladesh. Bangladesh J. Bioeth..

[14] Islam S., Emran G.I., Rahman E., Banik R., Sikder T., Smith L., Hossain S. (2021). Knowledge, attitudes and practices associated with the COVID-19 among slum dwellers resided in Dhaka City: A Bangladeshi interview-based survey. J. Public Health.

[15] Hossain M.A., Jahid M.I.K., Hossain K.M.A., Walton L.M., Uddin Z., Haque M.O., Kabir F., Arafat S.M.Y., Sakel M., Faruqui R. (2020). Knowledge, attitudes, and fear of COVID-19 during the Rapid Rise Period in Bangladesh. PLoS ONE.

[16] Ferdous M.Z., Islam M.S., Sikder M.T., Mosaddek A.S.M., Zegarra-Valdivia J.A., Gozal D. (2020). Knowledge, attitude, and practice regarding COVID-19 outbreak in Bangladesh: An onlinebased cross-sectional study. PLoS ONE.

[17] Rabbani M., Rahman S. Crisis of Communication during COVID-19, a Rapid Research. BRAC Institute of Governance and Development. https://bigd.bracu.ac.bd/study/crisis-of-communication-during-covid-19-a-rapid-research/.

[18] Benham J.L., Lang R., Kovacs Burns K., MacKean G., Léveillé T., McCormack B., Sheikh H., Fullerton M.M., Tang T., Boucher J.-C. (2021). Attitudes, current behaviours and barriers to public health measures that reduce COVID-19 transmission: A qualitative study to inform public health messaging. PLoS ONE.

[19] Vereen R.N., Lazard A.J., Frank S.C., Pulido M., Richter A.P.C., Higgins I.C.A., Shelus V.S., Vandegrift S.M., Hall M.G., Ribisl K.M. (2021). Motivations, barriers, and communication recommendations for promoting face coverings during the COVID-19 pandemic: Survey findings from a diverse sample. PLoS ONE.

[20] Sayeed Al-Zaman M. (2020). Healthcare crisis in Bangladesh during the COVID-19 pandemic. Am. J. Trop. Med. Hyg..

[21] Bakebillah M., Arif Billah M., Wubishet B.L., Nuruzzaman Khan M. (2021). Community’s misconception about COVID-19 and its associated factors: Evidence from a cross-sectional study in Bangladesh. medRxiv.

[22] Iorfa S.K., Ottu I.F.A., Oguntayo R., Ayandele O., Kolawole S.O., Gandi J.C., Dangiwa A.L., Olapegba P.O. (2020). COVID-19 knowledge, risk perception, and precautionary behavior among nigerians: A moderated mediation approach. Front. Psychol..

[23] Bruine de Bruin W., Bennett D. (2020). Relationships between initial COVID-19 risk perceptions and protective health behaviors: A national survey. Am. J. Prev. Med..

[24] Schoeni R.F., Wiemers E.E., Seltzer J.A., Langa K.M. (2021). Association between risk factors for complications from COVID-19, perceived chances of infection and complications, and protective behavior in the US. JAMA Netw. Open.

[25] IPA Monitoring Dashboard-Bangladesh. https://sites.google.com/poverty-action.org/mask-dashboard/dncc?authuser=0.

[26] Abaluck J., Kwong L., Styczynski A., Haque A., Kabir M.A., Bates-Jefferys E., Crawford E., Benjamin-Chung J., Benhachmi S., Raihan S. (2021). Normalizing Community Mask-Wearing: A Cluster Randomized Trial in Bangladesh.

[27] Ahmed N., Jahangir Rony R., Tuz Zaman K. (2020). Social distancing challenges for marginal communities during COVID-19 pandemic in Bangladesh. J. Biomed. Anal..

[28] Rahman M.S., Karamehic-Muratovic A., Amrin M., Chowdhury A.H., Mondol M.S., Haque U., Ali P. (2020). COVID-19 epidemic in Bangladesh among rural and urban residents: An online cross-sectional survey of knowledge, attitudes, and practices. Epidemiologia.

[29] Imtiaz A., Khan N.M., Hossain M.A. (2021). COVID-19 in Bangladesh: Measuring differences in individual precautionary behaviors among young adults. J. Public Health.

[30] Islam S., Islam R., Mannan F., Rahman S., Islam T. (2020). COVID-19 pandemic: An analysis of the healthcare, social and economic challenges in Bangladesh. Prog. Disaster Sci..

[31] Rahman M.R., Sajib H., Chowdhury I.M., Banik A., Bhattacharya R., Ahmed H. (2021). Present scenario of COVID-19 in Bangladesh and government preparedness for facing challenges. J. Adv. Biotechnol. Exp. Ther..

[32] Worldometer Coronavirus Testing Criteria and Numbers by Country. https://www.worldometers.info/coronavirus/covid-19-testing/.

[33] Islam M.T., Talukder A.K., Siddiqui M.N., Islam T. (2020). Tackling the COVID-19 pandemic: The Bangladesh perspective. J. Public health Res..

[34] Division of Disease Control (2020). National Guidelines on Clinical Management of Coronavirus Disease 2019 (COVID-19).

[35] Abedin M., Islam M.A., Rahman F.N., Reza H.M., Hossain M.Z., Hossain M.A., Arefin A., Hossain A. (2021). Willingness to vaccinate against COVID-19 among Bangladeshi adults: Understanding the strategies to optimize vaccination coverage. PLoS ONE.

[36] Karim M.R., Sah S.K., Syeda A., Faysol M.T., Rahman A., Islam K., Arefin A., Hossain A. (2020). Hand hygiene and personal protective equipment in healthcare settings during COVID-19 pandemic in Bangladesh. Bangladesh J. Med..

[37] Islam S.M.D.-U., Mondal P.K., Ojong N., Doza B., Siddique A.B., Hossain M., Mamun M.A. (2021). Water, sanitation, hygiene and waste disposal practices as COVID-19 response strategy: Insights from Bangladesh. Environ. Dev. Sustain..

